# Trends in the Regulation of Per- and Polyfluoroalkyl Substances (PFAS): A Scoping Review

**DOI:** 10.3390/ijerph182010900

**Published:** 2021-10-17

**Authors:** Nicole Marie Brennan, Abigail Teresa Evans, Meredith Kate Fritz, Stephanie Allison Peak, Haley Elizabeth von Holst

**Affiliations:** Battelle Memorial Institute, Columbus, OH 43201, USA; Brennan@Battelle.org (N.M.B.); Fritz@Battelle.org (M.K.F.); Peak@Battelle.org (S.A.P.); VonHolst@Battelle.org (H.E.v.H.)

**Keywords:** PFAS, policy, environment, regulation, analysis, exposure

## Abstract

Products containing per- and polyfluoroalkyl substances (PFAS) have been used for decades in industrial and consumer products. These compounds are persistent in the environment, bioaccumulative, and some are toxic to humans and other animals. Since the early 2000s, laws, policies, and regulations have been implemented to reduce the prevalence of PFAS in the environment and exposures to PFAS. We conducted a scoping literature review to identify how PFAS are regulated internationally, at the U.S. national level, and at the U.S. state level, as well as drivers of and challenges to implementing PFAS regulations in the U.S. This review captured peer-reviewed scientific literature (e.g., PubMed), grey literature databases (e.g., SciTech Premium Collection), Google searches, and targeted websites (e.g., state health department websites). We identified 454 relevant documents, of which 61 discussed the non-U.S. PFAS policy, 214 discussed the U.S. national-level PFAS policy, and 181 discussed the U.S. state-level PFAS policy. The drivers of and challenges to PFAS regulation were identified through qualitative analysis. The drivers of PFAS policy identified were political support for regulation, social awareness of PFAS, economic resource availability, and compelling scientific evidence. The challenges to implementing PFAS regulations were political limitations, economic challenges, unclear scientific evidence, and practical challenges. The implications for PFAS policy makers and other stakeholders are discussed.

## 1. Introduction

Since their invention in the 1930s and proliferation through the 1940s and 1950s, per- and polyfluoroalkyl substances (PFAS) have been used in a variety of industries including firefighting foam, aerospace technologies and consumer products. By replacing carbon hydrogen bonds with carbon fluorine bonds, scientists created one of the strongest compounds in organic chemistry; one that has proved to be resistant to heat, water, oil, and time [1,2,3,4,5]. Although PFAS have since been identified as persistent, bioaccumulative, and toxic, for decades they were lauded for their ability to protect firefighters and military personnel through enhanced firefighting foams that both extinguish fires and prevent reignition [6,7,8,9]. Their stability in hostile environments also made PFAS attractive to the aerospace, construction, and electronics industries [10,11,12]. However, the use of PFAS in firefighting foams and the discharge from industrial sites has led to widespread PFAS contamination in the global water supply [13,14,15,16]. People are also exposed to PFAS through products in everyday living, including non-stick cookware, and food wrappers, as well as stain-resistant clothes and furniture [17,18,19].

**Figure 1:**
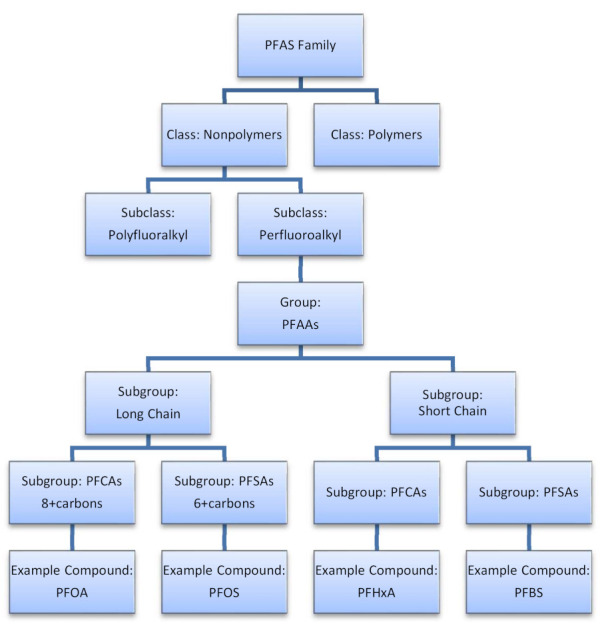
Overview of the major families of PFAS (per- and polyfluoroalkyl substances), including: PFAAs (perfluoroalkyl acids), 8 or more carbon PFCAs (perfluoroalkyl carboxylic acids/perfluoroalkyl carboxylates), PFOA (perfluorooctanic acid), 6 or more carbon PFSAs (perfluoroalkane sulfonic acids/perfluoroalkyl sulfonates), PFOS (perfluorooctane sulfonate/perfluorooctane sulfonic acid), 6 or fewer carbon PFCAs (perfluoroalkyl carboxylic acids/perfluoroalkyl carboxylates), PFHxA (perfluorohexanoate/perfluorohexanoic acid), 5 or fewer carbon PFSAs (perfluoroalkane sulfonic acids/perfluoroalkyl sulfonates), and PFBS (perfluorobutane sulfonate/Perfluorobutane sulfonic acid).

The exact number of unique PFAS compounds is unknown with estimates ranging between four and five thousand [1,7,20,21]. The categorization of the compounds and maintaining a consistent classification system is critical to studying the impacts of PFAS on health and the environment [22,23]. Some of the most discussed and regulated PFAS compounds are depicted in Figure 1. For example, the most commonly studied group of perfluoroalkyl substances are perfluoroalkyl acids (PFAAs). Across the scientific literature, PFAAs are generally broken into two groups, those with seven or more carbon-fluorine bonds (long-chain) and those with less than seven bonds (small-chain) [1,12,20,24,25]. While long-chain PFAAs are a small proportion of the overall PFAS family, long-chain perfluoroalkyl carboxylic acids (PFCAs) and long-chain perfluoroalkyl sulfonic acids (PFSAs) are the focus of international, domestic, and local scientific study and regulation due to their adverse impacts on the environment and human health [1,9].

**Figure 2:**
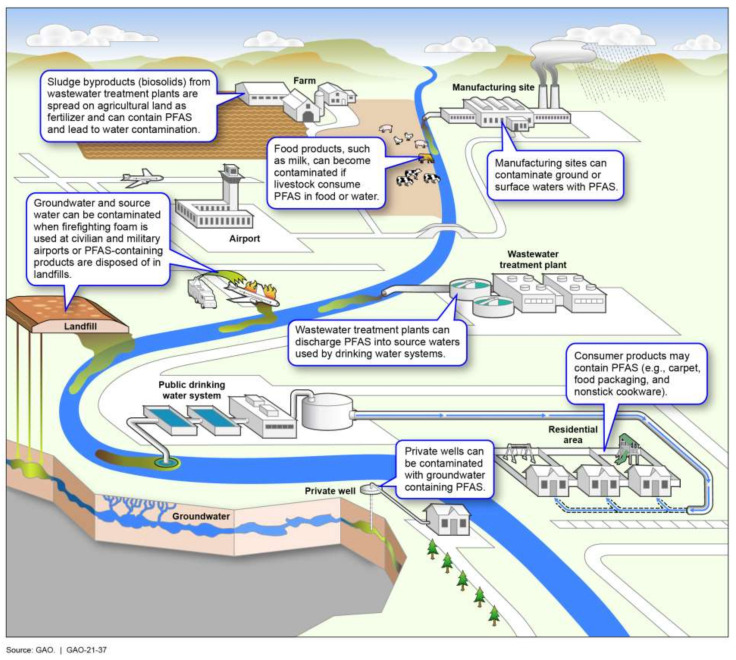
Overview of common pathways through which PFAS enter the environment. Originally published by the Government Accountability Office in report GAO-21-37: ‘Man-Made Chemicals and Potential Health Risks, EPA Has Completed Some Regulatory-Related Actions for PFAS’.

The chemical properties that make PFAS an asset for manufactured textiles, electronics, and other consumer products make them difficult to remove from the environment [26,27,28]. As depicted in Figure 2, PFAS enter the environment through many pathways. Due to their stable chemical structure, PFAS can enter and accumulate in the environment during production, use, and disposal [29,30,31]. These products remain present indefinitely in water supplies, seafood, and biosolids used in agriculture [29]. Furthermore, these substances accumulate in the systems of plants grown in contaminated soil [32] and animals that live in or drink contaminated water [33]. Their resistance to degradation, or physiological filtration has earned PFAS the moniker, “forever chemicals” [10,23,32].

Due to their environmental persistence and bioaccumulative nature, there is concern in the global community regarding the long-term impact of the exposure to PFAS on human health. The United Nations Organization of Economic Corporation and Development (OECD) released its hazard assessment for perfluorooctane sulfonic acid (PFOS) in 2002, which concluded that PFOS is “persistent, bioaccumulative, and toxic to mammalian species,” causing liver and thyroid cancer in rats [34]. The report indicated that PFOS exposure was also epidemiologically linked to bladder cancer in humans [34]. More recent work has shown epidemiological links between PFOS and higher levels of cholesterol, impaired glucose metabolism, increased body mass index, impaired thyroid function, infertility, and a higher prevalence of attention deficit hyperactivity disorder [35]. The European Food Safety Authority concluded that fish consumption was a significant source of exposure and estimated the bioconcentration factor to range from 1000 to 4000 depending on the geography and location in the food chain [36]. As a result of these and other investigations, PFOS sales were restricted to “essential uses” in Europe in 2006 [37] and PFOS was added to the Stockholm Convention on persistent organic pollutants in 2009 [38].

In the U.S., the concern around PFAS was initially driven by findings from a Centers for Disease Control and Prevention (CDC) investigation which found PFOS in the blood serum of nearly the entire population of the U.S. (in 2003, estimated to be 98%) [39,40]. The serum data collected from a nationally representative sample of Americans from 2013–2014 suggests the ongoing, near-universal exposure to perfluorooctanesulfonic acid (PFOS), perfluorooctanoic acid (PFOA), perfluorononanoic acid (PFNA), and perfluorohexane sulfonic acid (PFHxS) [41]. The bloodstream levels of PFOS and PFOA decreased after 3M and DuPont voluntarily phased out their production in the early 2000s [42,43]; however, both substances remain detectable in the bloodstreams of many Americans [44]. Animal studies and epidemiological investigations have linked the exposure to PFOS and PFOA to high levels of serum cholesterol, thyroid dysregulation, gestational hypertension, ulcerative colitis, and some cancers [45,46,47,48]. Children may be particularly at risk of negative health outcomes due to PFAS exposure; one systematic review found evidence that the prenatal and childhood exposure to perfluorinated alkyl substances was associated with dyslipidemia, impaired immune response (including reduced antibody response to vaccination), decreased kidney function, and a lower age of menarche [49].

Emerging international concern about the potential for PFAS to remain in the environment indefinitely spurred major manufacturers of PFAS to phase out the production of PFOS and PFOA in the early 2000s. Following this voluntary phase-out, PFAS levels in the bloodstreams of Americans decreased, suggesting the phase-out was effective in reducing human exposure [44]. However, these compounds were replaced with other PFAS, including shorter-chain homologues, such as perfluorobutane sulfonate (PFBS), and long-chain precursors, such as “GenX,” the trade name given to a processing technology used to generate chemicals that replace PFOA. Because short-chain PFAS were eliminated from the human body faster than their long-chain counterparts, they were believed to be a safer alternative to the long-chain PFAS that were phased out [50,51]. However, the US Environmental Protection Agency’s (EPA, Washington, DC, USA) 2018 draft toxicity assessments for GenX and PFBS found associations between these chemicals and harmful effects on the kidneys, immune system, liver, reproductive system, and organ development [52,53].

In an effort to reduce the exposure to harmful and potentially harmful PFAS, international bodies, individual countries, and local areas have undertaken efforts to limit PFAS manufacturing and reduce human exposure to PFAS. These efforts include restrictions on manufacturing and importing PFAS, setting maximum permissible levels of PFAS in food and drinking water, and regulating the disposal of products that contain PFAS. However, the standards are diverse and there is no consistent regulatory structure for this class of chemicals. There is also a disagreement on the appropriate level of regulation. While some stakeholders argue PFAS should be regulated at the compound level, others note it is impractical to regulate thousands of compounds and believe that scientific data on PFAS toxicity support regulating PFAS as a class due to their similarity in structure and stability [25,54,55].

The increasing scientific inquiry into the impact of PFAS on human health has spurred an increased interest in regulating PFAS at international, domestic, and local levels. Although there is a growing body of work relaying the types of policies under development or those that have been implemented at the U.S. national-level and across individual states [56,57], impactful policy development requires a holistic view of the system, including a historical perspective, and the reinforcing of restrictive feedback loops [58,59] affecting the implementation of policies, laws, and regulations. This paper seeks to create a narrative for PFAS policy developers seeking to understand the state of the PFAS regulatory system. We reviewed both peer-reviewed scientific literature (e.g., PubMed) and the relevant grey literature (e.g., government reports, targeted websites, trade journals) for data on PFAS policy discussion, development, implementation, and impact, at the international, domestic, and local levels. We outlined the progress of PFAS policy development and identified the key drivers of and challenges to PFAS policy implementation within the U.S. This approach allowed us to present a contextualized overview of the emerging issues, strategies, and solutions in this space. Our analysis intended to build on the PFAS dialogue and regulatory mechanisms already in progress. Synthesizing the current policy environment is a critical component to fill gaps in the scientific literature and build evidence-based regulatory requirements.

## 2. Methods

We performed a scoping review of the grey literature and peer-reviewed scientific literature to capture and synthesize contextual information on the history and landscape of PFAS regulation. The purpose of a scoping review is to provide an overview of the information available on a topic, which can include providing a broad overview of the topic and the content of information present in the literature, rather than providing an answer to a specific research question. Based on this search, we identified drivers of and challenges to regulating PFAS at the international, U.S. national, and state levels.

Our approach followed the grey literature review guidelines outlined by Godin et al. [60]. We defined grey literature as “information produced at all levels of government, academia, business and industry in electronic and print formats not controlled by commercial publishing, i.e., where publishing is not the primary activity of the producing body” [61,62]. The search included reviews of (1) grey literature databases (e.g., databases containing trade journals), (2) customized Google searches, (3) state and local legislation websites, and (4) a review of the content on targeted websites (e.g., websites maintained by groups that track environmental policy) [60,62,63]. To reduce the risk of selection bias, our review followed guidelines from the PRISMA Extension for Scoping Reviews (PRISMA-ScR) checklist including stating objectives, defining eligibility criteria, identifying information resources, the development of an electronic search strategy, and the synthesis of results [63].

### 2.1. Objective

The objective of our literature review was to identify the drivers of and challenges to PFAS policy implementation at the international, U.S. national, and U.S. state levels. All documents reviewed were assessed for their relevance to this objective.

### 2.2. Inclusion Criteria

Documents were considered relevant if they provided information about proposed or implemented PFAS policies or regulations. PFAS policy or regulation was defined as rules seeking to control the production, manufacturing, distribution, release, remediation, research, or safety guidelines of PFAS as established by an authority at the state, national, or international level. Relevant documents were added to an EndNote database. Inclusion and exclusion criteria are specified in Table 1.

### 2.3. Resources

#### 2.3.1. Peer-Reviewed and Grey Literature Databases

We conducted a systematic search of databases that captured grey literature and peer-reviewed scientific publications. The databases searched were PubMed (a database which captured citations from the biomedical literature, life sciences journals, and online books), SciTech Premium Collection (a database which captured science and engineering research, including grey literature), and Business Source Complete (a database which captured trade journals and business journals). Table 2 depicts the search strings used for the database search.

The authors reviewed the title, executive summaries, or table of contents for each document to make initial relevancy determinations. The full text of each relevant document was reviewed in detail.

#### 2.3.2. Keyword Searches

Google keyword searches were used to identify additional information. Numerous search strings were used (e.g., State + PFAS + Policy). Results from each keyword search were reviewed until content became redundant and/or no longer yielded relevant information (typically in the first one to two pages of search results).

#### 2.3.3. Targeted Websites

Additional resources were identified by searching the websites of organizations that regulates PFAS or monitored PFAS regulations. For example, PFAS state policy information was identified through state health departments, state legislatures, and bill tracking tool websites (e.g., National Conference on State Legislators, Washington, DC, USA, New York University State Energy & Environmental Impact Center, New York, NY, USA, United States Environmental Protection Agency, Washington, DC, USA, and The National Law Review, Western Springs, IL, USA).

#### 2.3.4. Synthesis

Document synthesis was initially divided into two parts: categorizing policies by government level (international, U.S. national, and U.S. state level), and identification of drivers and challenges. The authors reviewed the full text of relevant documents and categorized them based on their association with international-, U.S. national-, or U.S. state-level PFAS policies. Each document was then reviewed to construct the historical narrative of PFAS regulation and identify PFAS policy drivers and challenges. Drivers were defined as single or multi-level factors positively associated with the implementation of a policy related to PFAS or specific PFAS compounds. Challenges were defined as any single or multi-level factors hindering the initiation, advancement or approval of a policy related to PFAS or specific PFAS compounds. The literature search was completed on 31 December 2020.

## 3. Results

### 3.1. Documents

The database search identified a total of 214 unique documents, 141 of which were determined to be relevant. An additional 313 documents were identified through the keyword searches and targeted website reviews. In total, 61 relevant documents discussed international PFAS regulation, 214 discussed U.S. national-level PFAS regulation, and 181 documents discussed U.S. state-level PFAS regulation. The full database of sources used to develop this manuscript is provided as a Supplemental Material in Database S1. Although we had hoped to identify the drivers of and challenges to PFAS regulations outside the U.S., the articles retrieved about international PFAS regulations were primarily descriptive in nature regarding the policies themselves and did not discuss the drivers of and challenges to policy implementation. Thus, while we briefly describe some major international regulations, our analysis of the drivers of and challenges to PFAS regulations is only applicable to the U.S.

### 3.2. An Overview of PFAS Regulations

As the awareness of potentially harmful impacts of PFAS on human health grew within the scientific literature, legal scholarships, communities, PFAS guidelines, policies and regulations began to take effect in the early 2000s. In parallel with international and federal discussions, large PFAS manufacturers started voluntarily phasing out production of some long-chain PFAS. In addition to international treaties to prevent PFAS from entering the environment, signed in the early 2000s, some countries implemented additional national-, state-, and territory-level restrictions on PFAS. The US EPA released a Lifetime Health Advisory Limits for PFOS and PFOA in 2016 and advised municipalities to make consumers aware of PFAS-levels that exceeded those limits [64]. Following this release and absent national-level policies, the U.S. saw a rapid increase in the number of states that implemented policies to prevent PFAS pollution and protect consumers from exposure to these chemicals. Each government level faced drivers and challenges to PFAS regulation, which often included differing points of view presented by a diverse set of stakeholders. In the sections below, we briefly describe these major milestones in PFAS policies and regulations at the international, U.S. national and U.S. state levels, providing illustrative examples of the types of policies discussed, presented, and/or implemented.

#### 3.2.1. International and Non-U.S. PFAS Regulation

The Stockholm Convention on Persistent Organic Pollutants (POPs) is often cited as one the most significant pieces of collaborative international policy regarding environmental health and safety. The convention was adopted in 2001 and became international law in 2004 [65]. In 2009, the conference agreed to eliminate the production and use of PFOS under most circumstances, by adding it to the list of POPs within the convention [66]. A decade later, during the ninth meeting of the convention, members agreed to ban the use of firefighting foams containing PFOA and removed exemptions for the use of PFOS [67]. Currently, 152 countries have ratified the Stockholm Convention, including countries across all the inhabited continents [68]. According to the U.S. Department of State, “The United States signed the Stockholm Convention in 2001 but has yet to ratify because we currently lack the authority to implement all of its provisions. The United States participates as an observer in the meetings of the parties and in technical working groups” [69].

The 152 signatories of the Stockholm Convention include both developed and developing countries in Asia and South America [54]. A 2018 survey of 12 countries conducted by the International Pollutants Elimination Network found that, although some countries restricted PFOS in line with the Stockholm Convention, most PFAS substances were not regulated [70]. For example, although India became a party at the Stockholm Convention in 2006, no PFAS were regulated in the country at the time of the survey [70]. In Sri Lanka, the Stockholm Convention provision banning PFOS came into effect in 2010; however, other PFAS were not regulated [70]. China, also a signatory to the Stockholm Convention, is one of the largest producers and consumers of PFAS in the world [71]. Although the production of PFOS and PFOA were restricted in 2011 [72], China continued to allow the use of PFAS in firefighting foams [73]. Brazil is also a party of the Stockholm Convention [54]; however, a pesticide which degrades into PFOS (sulfluramid) continues to be produced and distributed there under a convention loophole [74] and has been linked to PFOS in soil, leaves, and coastal waters [75].

In 2006, the European Parliament and the Council of the European Union (EU) acknowledged “PFOS fulfil the criteria for classification as a very persistent, very bioaccumulative and toxic” chemical [37]. Subsequently, as part of the Registration, Evaluation, Authorization, and Restriction of Chemicals (REACH) regulation, the EU further noted “…that an unacceptable risk to human health and the environment arises from the manufacture, use or placing on the market of PFOA, its salts and PFOA-related substances” [76]. As of 2020, the European Commission estimates 100,000 sites are emitting PFAS [77]. The European Food Safety Agency (EFSA) has identified fish meat, fruit and fruit products, and eggs as common sources of dietary exposure to PFOA, PFNA, PFHxS and PFOS [78]. Based on existing data, they recommended a tolerable weekly intake of 4.4 ng/kg bw per week of these PFAS [78]. Concerned that short-chain PFAS were also bioaccumulative and persistent in the environment, the Commission published the European Chemicals Strategy on 14 October 2020 [79]. The strategy set a framework to ban all PFAS except those that cannot be replaced or were deemed essential to society [80,81]. Although these regulations were released, it is important to note that they are not currently enforced. Countries such as Norway, Germany, and Sweden played pivotal roles in the development of these regulations, with Norway and Germany initiating the addition of PFOA to REACH, and in 2017, Sweden and Germany asked the EU to consider adding another PFAS compound, PFHxS, to the list of substances of very high concern [77]. Furthermore, as early as 2018, Germany began submitting proposed restrictions on PFHxA [82]. In 2019, the member state committee agreed with the classification proposed by the Dutch identifying hexafluoropropylene oxide dimer acid (HFPO-DA)/GenX compounds as substances of very high concern [83].

Other countries, including Canada, used a combination of government regulations and voluntary agreements with industry to limit PFAS pollution [84]. In the early 2000s, Canada began taking actions to develop rules and regulations for PFAS, releasing a risk management strategy for PFOS in 2006 [85]. *The Environmental Performance Agreement respecting Perfluorinated Carboxylic Acids (PFCAs) and their Precursors in Perfluorinated products sold in Canada*, an agreement negotiated between Environment and Climate Change Canada and PFAS producers, Arkema Canada Inc., Asahi Glass Company LTD, Clariant Canada Inc., and E.I. du Pont Canada Company, came into effect in 2015, at which time the participating companies had eliminated the production of PFOA, long-chain PFCAs and their precursors [86].

Australia has taken measures to reduce human exposure to PFAS and limit PFAS pollution at the state and local level rather than at the national level. Although Australia is a party of the Stockholm Convention, its ratification is subject to an individual analysis of chemicals listed in Annex A, B, or C of the convention [87], which has delayed the adoption of the restrictions outlined in the convention. In a memo about the management of PFAS, the Australian Department of the Environment and Energy reported “environmental risk management has been a gap in Australia’s regulatory system…” and noted that state and territory environmental agencies have worked actively to fill this gap [88]. Instead of creating national regulations, the Australian government has worked to develop national standards which could be implemented by states and territories. For example, Food Standards Australia New Zealand developed a set of guidance values for food including tolerable dietary exposure levels for PFOS, PFOA, and PFHxS and tolerable daily intake levels for water [89]. Individual territories were responsible for conducting site investigations and mandating remediation efforts where necessary. Some states implemented bans that were intended to prevent PFAS pollution. For example, in 2019, a Queensland ban prohibiting the use of firefighting foams containing PFOS and PFOA came into effect [90]. South Australia implemented a similar ban that came into effect in 2018 [91]. However, when asked if New South Wales would pursue a similar ban, State Environment Minister, Gabrielle Upton, stated that “This government cannot ban PFAS … The responsibility for that lies directly at the feet of the federal government …” [92].

#### 3.2.2. PFAS Regulations at the U.S. National-Level

The U.S. Environmental Protection Agency (EPA) has the authority to regulate PFAS through the Toxic Substances Control Act (TSCA), the Safe Drinking Water Act (SDWA), the Comprehensive Environmental Response, Compensation, and Liability Act (CERCLA), and other regulatory authorities. The EPA’s efforts to control the proliferation of PFAS have focused on PFOS, PFOA, and other PFAS which have been voluntarily phased out by industry leaders [93]. The EPA’s regulation of PFAS began with two Significant New Use Rules (SNURs) published in 2002, which required manufacturers to provide the EPA with a notification about the manufacture or import of 13, and later 75, PFAS chemicals being voluntarily phased out by 3M from 2000–2002 [94,95]. The EPA negotiated with PFAS manufacturers, 3M and DuPont, to produce memoranda of understanding in 2003, which detailed their plans to stop using these substances [42,43]. In 2006, the EPA invited eight major PFAS producers to join the PFOA Stewardship Program, which pledged to reduce 95% of their facilities’ PFOA emissions by 2010 [96].

The EPA’s regulation of PFAS through the SDWA began in 2009, when the EPA released provisional health advisories for PFOS and PFOA. Between 2013 and 2015, the EPA monitored levels of PFOS, PFOA, and PFNA in drinking water supplies as part of their unregulated contaminant monitoring [97]. In 2016, the EPA issued a lifetime drinking water health advisory level of 70 parts per trillion (ppt) for PFOS and PFOA [64], a level which was between seven and ten times greater than the levels recommended in the Agency for Toxic Substances and Disease Registry’s (ATSDR) 2018 draft, *Toxicological Profile for Perfluoroalkyls* [98]. Health advisory levels are not enforceable standards, rather, they are values developed to assist federal, state, tribal, and local officials in their efforts to protect public health where these chemicals are found in drinking water supplies. In February 2019, the EPA released their *PFAS Action Plan*, a document intended to outline the steps the EPA was taking to protect public health by addressing PFAS [99]. The plan indicated that the EPA was moving forward with the regulatory process to set Maximum Contaminant Levels (MCLs) for PFOA and PFOS. Furthermore, it indicated that EPA would pursue the process for designating PFOA and PFOS as hazardous substances under CERCLA or Superfund, which could hold companies liable for the cleanup of hazardous substances that they released into the environment. However, the EPA has not yet completed the regulatory processes to set MCLs for PFOA and PFOS or to designate PFOA and PFOS as hazardous substances under CERCLA.

Federal lawmakers and environmental activists expressed a frustration with the EPA’s response to widespread PFAS contamination. Dozens of bills were introduced in Spring 2019 and throughout the 116th Congress which sought to expedite the EPA’s regulatory timelines. For example, S. 1473, the Protect Drinking Water from PFAS Act of 2019, would have required the EPA to issue a MCL goal, as well as a national primary drinking water regulation, for some PFAS [100]. House Resolution (H.R.) 2605, or the Protect Release Of Toxic Emissions, Contamination, and Transfer (PROTECT) Act of 2019, would have required the EPA to issue a final rule adding PFAS to the list of hazardous air pollutants within 180 days [101]. H.R. 2596, the Protecting Communities from new PFAS act, would have required all manufacturers to notify the EPA about all PFAS manufacturing activity, including PFAS that were not listed on the EPA inventory list [102]. Many of these bills were incorporated into H.R. 535, the PFAS Action Act of 2019 [103], which passed in the House on 1 January 2020. This bill was a comprehensive package of strategies to regulate PFAS chemicals, clean up contamination, and protect public health. Although Senate Bill 1507 would have required the EPA to include certain PFAS on the toxics release inventory [104] and S. 1372 would have compelled federal agencies to enter into cooperative agreements to remediate PFAS [105], no comprehensive legislation package such as the PFAS Action Act of 2019 was introduced in the Senate during the 116th Congress. Instead, the Senate moved to include PFAS-related provisions in the Water Resources Development Act, a regularly updated act which authorizes the Army Corp of Engineers’ water infrastructure projects [106]. However, PFAS-related provisions were removed from the version of the bill which ultimately passed in the House [107].

The Department of Defense (DOD) has also acted to reduce the exposure to PFAS for individuals residing near active and inactive military installations, many of which have used aqueous film-forming foam (AFFF) which contains PFOS, and in some versions, PFOA to extinguish petroleum-based fires. A 2017 Government Accountability Office (GAO) report found that the DOD had spent approximately USD 200 million on investigating and responding to PFAS contamination, had identified 401 installations where PFAS may have been released, and took actions to address the contamination at 32 locations [108]. More recent military documents show that as many as 600 active and inactive military installations could be contaminated by PFAS, and groundwater levels well above 1 million ppt were found at 14 installations [109]. Federal leaders have expressed frustrations about the DOD’s slow response to PFAS contamination near military sites. The 2020 National Defense Authorization Act (NDAA), which provides funding for all DOD programs, included a number of provisions related to PFAS, including requirements to phase out the use of PFAS in firefighting foams, providing blood tests to military firefighters, addressing PFAS contamination in water supplies due to military activity, promoting PFAS water contamination monitoring, requiring the EPA to list PFAS chemicals under TSCA, and restricting the use of PFAS in military food packaging [110]. The 2021 NDAA, which passed the House on 21 July 2020, offered additional funding for PFAS monitoring and remediation efforts including USD 1.5 billion to support PFAS cleanup efforts, USD 150 million to support research on PFAS remediation, and USD 15 million for the continuation of CDC’s study on the health effects of PFAS groundwater contamination at military bases [111].

In July 2020, the US Food and Drug Administration (FDA) announced that they had reached an agreement with three manufacturers who used PFAS that contained 6:2 fluorotelomer alcohol (6:2 FTOH) for grease-proofing in paper and paperboard food packaging [112]. The three manufacturers agreed that, in January 2021, they would begin a three-year phase-out period for the sale of substances that contained 6:2 FTOH [112]. The FDA lauded the agreement as a solution that “balances uncertainty about the potential for public health risks with minimizing potential market disruptions to food packaging supply chains” [112].

#### 3.2.3. PFAS Regulations at the U.S. State-Level

In light of the absent national drinking water standards for PFAS, as of 1 May 2020, nine U.S. states developed drinking water standards or guidance values for PFOS and PFOA which were more stringent than those issued by the EPA [113]. Some states developed guidelines for other PFAS as well (e.g., PFNA, PFHxS) based on data published by ATSDR and the European Food Safety Authority [113]. However, the variability of these policies illustrated a fragmented network of PFAS laws targeting diverse sources of exposure including water, foams, and food. The California Assembly Bill (AB) 756, passed in 2019, allowed the state board to order public water systems to monitor for PFAS, and subsequently required the water system to remove a water source or provide a public notice if levels were above a prescribed amount [114]. In the same year, Vermont passed ACT 21 (Senate Bill 49), which laid out strict requirements for the government monitoring of PFAS levels in municipal water systems [115]. While the bill did not regulate PFAS as a class, it required that all public community water systems in the state complete an initial monitoring for the presence of PFAS and laid out requirements for the further monitoring based on PFAS detection. Further, if PFAS were detected at levels above 20 ppt, the health advisory level set by the state, public water systems were required to issue a “do not drink” notice to all users of the water system until treatment was complete [116].

California subsequently expanded the regulations for firefighting foam. In September 2020, California prohibited the manufacture and sale of firefighting foam containing PFAS and requiring manufacturers of protective firefighter equipment to disclose if PFAS were in their products. The violators of this law could be fined up to USD 10,000 after the first offense [117]. In addition to these bills, California adopted four additional PFAS-focused bills.

Similarly, in May 2020, the Maryland state legislature submitted a bill to the governor, which prohibited the use of Class-B firefighting foam that contained intentionally added PFAS chemicals for testing or training purposes, by October 1, 2021. The violation penalties for this bill ranged from 500–1000 USD per penalty. The legislation clearly delineated that the bill did not restrict “the discharge or use of class B fire-fighting foam that contain intentionally added PFAS chemical in emergency fire-fighting or fire prevention operations” [118]. In 2018, Washington passed a state-level bill, titled *Concerning the use of perfluorinated chemicals in food packaging*, which prohibited the use of PFAS in food packaging if a safer alternative could be identified. The use of PFAS for specific food packaging applications was prohibited, beginning two years after a report was released, identifying a safer alternative [119]. Similar bills were also adopted in Maine and proposed in 14 additional states such as New Hampshire, Ohio, Arizona, Virginia, and New Jersey [57,120,121,122].

In addition to laws which seek to directly reduce human exposure to PFAS, some states have created legislation that provides funding for testing sites suspected of PFAS contamination or have undertaken other efforts to hold companies that release PFAS into the environment accountable for clean-up costs. For example, the Tennessee Department of Environment and Conservation is currently conducting a statewide effort to sample public drinking water systems for the presence of PFAS [123]. If PFAS are identified in the water system, the state will seek to identify the sources of the water contamination.

Other tactics have included establishing coalitions and taking legal action against PFAS suppliers. In Michigan, the Executive Directive 2017-4 established the PFAS Action Response Team (MPART) to act as a multi-agency collaborative working to locate and investigate where PFAS contamination originated in the state and to protect the state’s water supply from PFAS [124]. In Vermont, rather than passing legislation, the state filed a lawsuit against Saint-Gobain Performance Plastics after finding that wells in the area near a closed plastics manufacturing plant had been contaminated with PFOA [125]. The state eventually reached a settlement with Saint-Gobain in which they agreed to pay for water line extensions to approximately 200 homes whose well water had been contaminated [126].

With more than half of the states working on PFAS legislative efforts, it is important to not only understand the content of the legislation, but the variables which influence policy adoption.

### 3.3. Drivers of and Challenges to PFAS Policy Implementationin the U.S.

The drivers and challenges to PFAS policy implementation in the U.S. were categorized into commonly used policy analysis evaluation variables: political, social, economic, scientific, and practical. An overview of each major theme is provided in Table 3.

#### 3.3.1. Drivers of PFAS Policy Implementation

The synthesis of the peer-reviewed literature and policy documents identified variables characterized as drivers of PFAS policy, which could be divided into four variables and eight sub-variables. These variables are defined in Table 4 and discussed in the sections below.

##### Political Support

Perceived Regulatory Need. Several states, including California, Michigan, and Ohio, cited the lack of federal regulatory action as a driver for state policy [127,128,129,130,131]. In the Connecticut Interagency PFAS Action Plan, the committee lauded the EPA’s PFAS Action Plan; however, also noted that the timelines within the plan were too long, “Given the long time frame for future federal regulations governing PFAS exposure, use and disposal, state leadership in this regard is crucial.” Accordingly, on 8 July 2019, Governor Ned Lamont established the Connecticut Interagency PFAS Task Force with the goal of educating residents about the potential risks associated with PFAS and implementing the appropriate safeguards [132].

Political Collaboration. Within the U.S., there is a general acceptance between political parties that PFAS issues need to be addressed through policies, science, and appropriations; however, there is a divide on the approach and speed of advancing new regulations to best minimize the effects of PFAS on citizens and the environment. In general, politicians who are members of the Democratic party are likely to be more aggressive in advocating for comprehensive PFAS regulations with shorter timelines, while members of the Republican party prefer a more cautious and deliberate approach in advancing new regulations [133,134,135]. However, bipartisan collaboration has occurred and may be more likely to lead to policy implementation than bills proposed by members of one party alone. For example, at the national level, although many bills to address PFAS were introduced in the House during the 116th Congress, the only PFAS-specific bill which passed was the bipartisan PFAS Action Act [136]. Bipartisan coalitions also worked to ensure that PFAS provisions became part of the 2019 defense omnibus spending bill [137].

The bipartisan collaboration to pass PFAS policies also occurred at the state-level. For example, in February 2020, Wisconsin state senators from both parties worked together to introduce a set of proposals that included creating emergency rules for setting drinking water, ground water, and air standards for PFAS [138,139]. In New Hampshire, a bill to offer loans to towns whose water supplies were unable to meet the stringent state standards set in SB496 passed with unanimous support [140,141].

Political Leader Advocacy. The advocates within the political party in power help to drive PFAS policy and guidelines at the state-level. In 2019, Ohio Republican Governor Mike DeWine worked to develop a PFAS action plan by leading collaboration efforts between the Ohio Environmental Protection Agency (Ohio EPA) and the Ohio Department of Health (ODH). The objective of the action plan was to remediate threats to private and public drinking water [127]. The action plan delineated objectives and laid the foundation for the Ohio PFAS Action Plan Interactive Dashboard and Map showing PFAS sampling information from across the state [142].

Similarly, in 2019, Michigan Democratic Governor Gretchen Whitmer requested that the Michigan PFAS Action Response Team (MPART) inform the rulemaking process for the health-based values of PFAS drinking water [143]. MPART was comprised of seven state agencies (e.g., Michigan Depart of Environment and Michigan Department of Transportation), and assigned an executive director who worked directly with three advisory councils, including a science advisory workgroup, a local health department advisory council and a citizens advisory workgroup [144]. In line with recommendations from MPART, Michigan passed PFAS drinking water standards noted to be more restrictive than the EPA guidance [145].

Reinforcing Legislative Feedback Loops. The regulations and recommendation reports from states actively implementing PFAS regulations, are being used as drivers for policy development in other states across the country. The Executive Summary from “Health-Based Drinking Water Value Recommendations for PFAS in Michigan” authored by MPART’s Science Advisory Task Force, was cited as supporting evidence within Alaska’s Senate Bill 176, and House Bill 240: Regulate PFAS Use Fire/Water Safety [146]. The documents supporting Vermont’s legislation to regulate PFAS in water included a Bloomberg article, in which the submitter noted that it “includes a state-by-state summary of efforts to regulate PFAS chemicals. There is lots of helpful information here…” [147]. When discussing *AB756*, the California Committee on Environmental Safety and Toxic Materials cited several scientific studies conducted across the U.S. and Europe [131].

Feedback loops also occurred at the state level. In support of Maryland’s legislation restricting the use of PFAS in firefighting foam, a Baltimore County executive submitted a letter in favor of the legislation noting, “This bill is one of Baltimore County’s top legislative priorities this session for a simple reason: PFAS are in our waters right now. They have been detected in enormous quantities in the Patuxent River and Chesapeake Beach. We now ask the State to prioritize this essential legislation as well. We cannot afford to let PFAS levels rise more than they already have” [118].

##### Social Awareness

Increases in the social awareness of PFAS and their potential danger is another driver of PFAS policy. The movies such as “The Devil We Know” and “Dark Waters,” and the newspaper articles that are the origin of their plot points, introduced many Americans to PFAS by providing compelling narratives of legal battles between PFAS manufacturers and people who had been negatively affected by PFAS [21,92,148,149]. Following the publication of a news story about the presence of GenX in North Carolina waterways, the North Carolina state legislature passed a bill providing USD 5 million in funding to establish a multi-university consortium for PFAS testing [150]. This increased social awareness has resulted in grassroots campaigns such as the National PFAS Contamination Coalition (NPCC), whose stated top priority is “to advocate for national regulation of PFAS as a class with a MCL of 1 part per trillion (ppt) or less” [151].

In Michigan, the social awareness and public concern about water quality has remained high following a 2014 crisis in which lead-contaminated water was supplied to approximately 9000 children [152]. In 2017, the MPART was created as a temporary body to investigate PFAS contamination in the state, which was later made permanent by the Executive Order 2019-3. The organization conducted PFAS monitoring in the state’s waterways and found evidence that commercial AFFF was responsible for the high concentrations of PFAS found at sites throughout the state. Drawing on MPART’s findings, State Attorney General Dana Nessel filed lawsuits in both state [153] and federal courts [154] against 17 companies that manufactured, sold, and distributed AFFF made with PFAS, including DowDuPont, 3M, and Chemours, aiming to recover the costs of PFAS identification, monitoring, and remediation efforts. The lawsuits allege that the manufacturers knew for decades that PFAS in commercial AFFF posed substantial threats to the environment and public health, but intentionally hid this information from the public. Based on MPART’s guidance, Michigan recently passed some of the most stringent groundwater limitations for PFOS and PFOA in the country [155]. Furthermore, the state set MCLs for PFNA, PFHxA, PFHxS, PFBS, and HFPO-DA [156].

##### Economic Resource Availability

Federal investments in EPA initiatives have helped to drive state-level PFAS monitoring and remediation efforts. For example, the EPA provided technical assistance to states in its fourth region, which included Alabama, Florida, Georgia, North Carolina, and South Carolina. As part of these efforts, the EPA collaborated with Georgia and Alabama to conduct a study of PFAS levels in Alabama’s Weiss Lake and its tributary systems. Levels of PFAS of up to 375 ppt were identified in this area’s waterways and were linked to carpet factory waste and the use of firefighting foam at nearby air force bases [157]. The data collected by the EPA and local governments will inform local monitoring and remediation efforts, as well as the knowledge base regarding the fate and transport of PFAS, which will support the EPA’s future rulemaking efforts [158].

In Florida, the EPA is working with the Florida Department of Environmental Protection (FDEP) and the Navy to address PFAS contamination at two Naval air stations [159]. Additionally, EPA worked with the state to improve the lab capacity for PFAS testing and to identify the appropriate test methods for PFAS in biosolids.

In North Carolina, the EPA analyzed thousands of water samples collected from the Cape Fear River, where residents were concerned about GenX entering the water from a Chemours plant [160]. These data helped to inform the North Carolina Department of Environmental Quality’s (NDEQ) decision to issue a no discharge limitation order for the site [161]. In 2019, NDEQ reached a consent order requiring Chemours to invest in technology to prevent PFAS emissions through the air and water and to fund the compliance testing of nearby waterways [162].

##### Compelling Scientific Evidence

Evidence linking specific PFAS to adverse health outcomes is often the impetus for PFAS regulation. Nearly every piece of legislation reviewed had supporting documents with a synopsis of the current findings relating PFAS to poor health outcomes [130]. Section 1 of the Colorado Act: *Concerning the use of perfluoroalkyl and polyfluoroalkyl substances, and in connection therewithin, making an appropriation,* states, “The historic use of perfluoroalkyl and polyfluoroalkyl substances, known as PFAS chemicals, in Class B firefighting foams has contaminated the drinking water of nearly 100,000 Coloradans…. PFAS chemicals do not break down in the environment and are toxic to people and wildlife at very low levels. Ingesting even small amounts can cause cancer and other serious health problems. Exposure to PFAS chemicals is linked to kidney and testicular cancer, thyroid problems, pregnancy complications, high cholesterol, and immune system disorders. Firefighters and first responders are exposed to these chemicals at work and nearly every American has measurable amounts in their bodies” [163].

Many policies reference the 1998 notification 3M sent by the EPA, which stated that PFOS accumulated in the blood stream and provided data showing liver damage in rats following exposure [164,165]. The data reported in these documents motivated 3M to voluntarily discontinue the production of PFOS and PFOA in 2000 [42,43].

In 2014, the European Chemical Agency Risk Assessment Committee classified PFNA as a hazardous substance, characterizing it as a suspected carcinogen known to be toxic to reproduction [166]. These findings were cited by the New Jersey Drinking Water Quality Institute’s Health Effects Subcommittee report, which recommended health-based MCLs for several PFAS [167]. In 2018, the EPA released its draft toxicity statements for GenX and PFBS [53,168]. These reports concluded that the liver was particularly sensitive to GenX and that the thyroid was particularly sensitive to PFBS. Michigan’s MPART considered these reports when developing the health-based values used to support the MCLs for the state [169].

The author of California’s laws prohibiting the manufacture, sale and distribution of AFFF containing PFAS noted: “Firefighters already face greater risks of cancer and other health problems than the general population due to exposure related to their vital work. Firefighting protective gear also contains PFAS, so there is an exposure from both the gear and the firefighting foam. The elevated levels of PFAS chemicals have been documented in the bodies of firefighters, putting them at greater risk of harm from the health effects associated with PFAS, including cancer” [117].

#### 3.3.2. Challenges to PFAS Regulations and Policies

The synthesis of peer-reviewed literature and policy documents identified variables characterized as challenges to PFAS policy, which could be divided into four variables and eight sub-variables. These variables are identified in Table 5 and discussed in the sections below.

##### Political Limitations

Restrictive Legislative Feedback Loops. The unclear delegation of responsibility for preventing and remediating PFAS can create feedback loops that delay PFAS policies and regulations from being implemented. At the U.S. national-level, the EPA has so far only released non-enforceable lifetime health advisory levels for PFOA and PFOS of 70 ppt, individually or combined [170]. Although the EPA has indicated its intention to develop enforceable limits for PFOS, PFOA, and other PFAS levels in drinking water under the SDWA, there are no data available about what those limits will be or the timeline for when they will go into effect [171]. Some legislators have expressed frustration with the EPA’s timeline for implementing these regulations [172,173].

U.S. state-level laws, policies, and regulations have been challenged by stakeholders who believe that PFAS regulations should be handled at the U.S. national level. The public testimony on Maine’s restriction of PFAS in food containers called into question Maine’s ability to circumvent a process already overseen by the FDA. One stakeholder from the Maine Grocers and Food Producers Association noted, “We are testifying in opposition to this proposal because it would yield federal oversight of acceptable packaging currently governed by the US Food & Drug Administration to the Maine Department of Environmental Protection. The US FDA oversees for the regulation of direct and indirect-additives use in food contact packaging materials and is based on extensive testing for each specific material and should be done at the federal level” [174]. This sentiment was shared by stakeholders from the FluoroCouncil and the Association of Washington Business, who testified against a similar bill in Washington, stating “This would restrict all PFASs unnecessarily, without actual attention paid to use. The FDA strictly regulates food packing. We think this is premature because of the chemical action plan Ecology is currently conducting. The terms in the bill are vague or undefined. We cannot control what kind of food packaging is brought into the state. There needs to be more than a safer alternative. We need multiple alternatives to be available. Food costs are sensitive and changing food packaging options may affect costs” [175,176].

Regulatory Limitations. In some cases, a lack of federal guidance and regulation prevents states from implementing their own PFAS laws, policies, and regulations. In a survey of states’ drinking water and groundwater guidelines conducted by the Environmental Council of States (ECOS), eight of the twenty-three states surveyed indicated that they had not implemented any state-level PFAS guidelines [177]. Five of these states indicated that the state had restrictions prohibiting them from setting guidelines for drinking water or groundwater guidelines that were more restrictive than those set by the federal government [177].

Differing Priorities. Addressing PFAS is not a legislative priority in every state. For example, the head of the Utah Department of Environmental Quality, stated that, although some states order that their drinking water is tested for PFAS, Utah does not need to test for PFAS because the state is not home to PFAS manufacturers [178]. Further, the official noted that Utah was at an advantage in protecting its citizens from PFAS because their drinking water was derived from the snowpack stored in the mountains and did not come from rivers with wide watersheds. A spokesman for Nebraska’s Department of Environmental Quality expressed a similar sentiment, stating: “We haven’t taken a regulatory lead in this area to date, as preliminary results indicate that Nebraska does not appear to have PFAS issues to the extent that some other areas in the nation are experiencing” [179] He went on to explain that the state “will continue to stay involved in these emerging issues” [179].

##### Economic Challenges

The high costs of PFAS monitoring and remediation efforts are a significant challenge to PFAS regulations and policies. The possible costs include implementing testing and monitoring programs, providing alternative water supplies to people with contaminated drinking water, retrofitting water and sewage treatment plants to filter out PFAS, site cleanup in areas where PFAS have been used, and more. The Congressional Budget Office estimated that the cost of bill S. 1507, which would have authorized grants for states to address “emerging contaminants” in drinking water, including PFAS; to direct the U.S. Geological Survey to set PFAS concentration standards in groundwater; to direct the EPA to study, monitor, and regulate PFAS in drinking water; and to establish a multiagency initiative to study emerging contaminants, would be USD 715 million between 2020 and 2024 [180].

The high costs may cause some state health departments and concerned citizens to struggle to convince legislators to invest in PFAS remediation efforts. For example, the New Hampshire Department of Environmental Resources proposed drinking water standards for the state that would set MCLs for PFOS or PFOA and PFOS combined at 70 ppt, 38 ppt for PFOS alone, 85 ppt for PFHxS, and 23 ppt for PFNA [181]. They estimated that these standards would initially cost the public water system between USD 2.2 million and USD 8 million and indicated that the state would make grants available to cover costs. However, the court blocked the standards from coming into effect, stating that a more thorough cost–benefit analysis was needed to justify the expense that the standards would pose for taxpayers [182]. In New York, the Governor made an emergency declaration that established PFOA as a hazardous substance and classified Hoosick Fall and Petersburg as State Superfund Sites. These designations were necessary to make emergency funding available for PFAS water contamination cleanup efforts originating from a Saint-Gobain Performance Plastics Site [183]. However, the negotiations between Hoosick Falls and Saint-Gobain on the cost of cleanup and remediation efforts have been the subject of intense debate [184]. Other states have struggled to estimate remediation costs. Benchmarking costs based on what other states have experienced can be difficult due to the variability in remediation objectives [21].

##### Unclear Scientific Evidence

Scientific Ambiguity. Although there is robust evidence from human and animal studies that exposure to PFOS and PFOA (which remain widely present in food and the environment) can cause negative health outcomes, there are over 4700 chemicals in the PFAS family and toxicity data are limited or unavailable for most of them [10,21,99,185]. Long-chain PFAS are currently thought to present a greater toxicity than shorter-chain PFAS because they are more difficult to metabolize [186,187]. Thus, many PFAS producers switched from using long-chain PFAS to short-chain PFAS. However, less research has been conducted on the toxicity of shorter-chain PFAS [188]. Emerging evidence suggests that short-chain PFAS can also pose significant risks to human health. For example, the EPA’s draft toxicity reports found evidence in animal studies that GenX was associated with negative health effects in the kidneys, blood, immune system, and the liver and PFBS was associated with negative effects in the thyroid, reproductive organs, developing fetuses, and the kidneys [189].

Though European countries allow for regulation based on the precautionary principle (i.e., proactively endorsing mitigation measures when evidence is uncertain), U.S. regulatory agencies typically require substantial data showing significant risks before regulations can be implemented [190,191]. At the U.S. national level, the statutory authorities best suited to PFAS regulation, such as the SDWA, require toxicity assessments for each chemical being regulated. Many researchers have recommended that PFAS should be regulated as a class rather than as individual chemicals [9,25]. However, some argue that this would not be appropriate because each PFAS has a unique chemical structure and they exist in many compounds that can break down differently in the environment [192]. EPA has determined that 160 PFAS chemicals may warrant inclusion on the Toxic Substances Release Inventory; however, this is far less than the total number of PFAS currently in use [193].

At the state-level, some states have not implemented PFAS regulations or policies because it is not yet clear how PFAS compounds have affected their residents. Although the PFAS pollution in North Carolina became well known as a result of stories covering GenX in the Cape Fear Basin, additional research has been called for while the North Carolina Department of Health and Human Services (DHHS) sets a provisional health advisory of 140 ppt for GenX. The North Carolina state legislature provided USD 5 million to the NC Policy Collaboratory to fund a multi-university PFAS research initiative called the PFAS Testing Network (PFAST Network). Recently, the North Carolina Attorney General announced plans to further identify the manufacturers and other parties responsible for PFAS contamination in the state [194].

Similarly, although Tennessee has no PFAS regulations in place, the Tennessee Department of Environment and Conservation (TDEC) is currently conducting an assessment of the state’s water system to identify PFAS levels in drinking water, with results expected in 2021 [195]. TDEC has indicated these results may be used to identify places where remediation is needed or to identify which PFAS should be regulated by the state. Although the Montana Department of Environmental Quality recently added PFOS and PFOA to the state’s Circular DEQ-7 Montana Numeric Water Quality Standards at 70 ppt [196], objectives in the state’s PFAS action plan include “Consider PFAS in Source Water Protection Plans” and “Pursue preventive measures (legislation, regulation, permitting)” [197].

##### Practical Challenges

Practical Challenges. Policy makers seeking to implement PFAS policies and regulations are faced with technical challenges in attaining the information needed for monitoring and enforcement efforts. Capturing and analyzing PFAS samples that are appropriate for regulation is challenging because the widespread use of PFAS means that many of the materials normally used to collect field samples and to complete laboratory operations are often contaminated with PFAS [198]. For example, the polytetrafluoroethylene products (tubing, sample containers, and sampling tools) used to collect samples can contain PFAS. The consumer goods often brought to testing sites can also be a source of sample contamination [11]. The EPA published its validated Method 537 for monitoring the levels of 14 different PFAS in water samples in 2009. This method was later expanded to monitor four additional PFAS, including GenX [11]. Although some states have developed their own guidelines for sampling and analyzing PFAS [199], the development of such guidelines may exceed the capacity of some state health departments.

Both Washington and Maine legislation note that alternatives to PFAS must be found. In Maine, individuals concerned about prohibiting the use of PFAS in food packaging said that the state needed to determine that a safer alternative is available in a sufficient quantity and at a comparable cost [122]. Washington legislation restricting the use of firefighting foams which contain PFAS included a clause to provide airports additional time to secure firefighting foams without intentionally added PFAS that meet federal requirements if they were not readily available when the legislation was scheduled to take effect [200].

**Figure 3:**
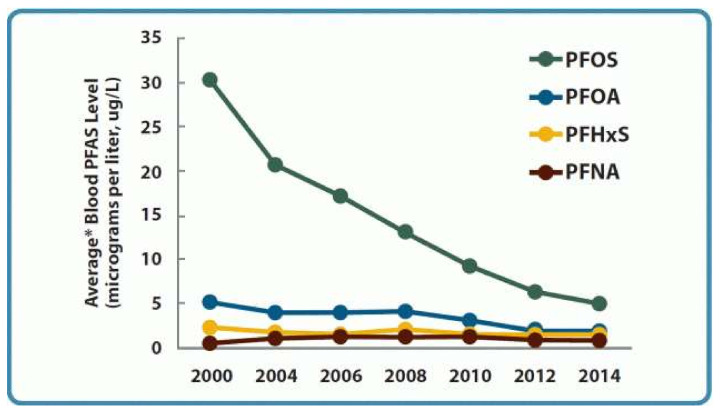
Blood Levels of the Most Common PFAS in People in the United States from 2000–2014. Originally published in Fourth Report on Human Exposure to Environmental Chemicals, Updated Tables, (January 2017). Atlanta, GA: U.S. Department of Health and Human Services (HHS), Centers for Disease Control and Prevention (CDC). This figure is freely available on the agency’s website and its use does not imply endorsement of this manuscript by CDC, HHS, or the United States Government. PFAS (per- and polyfluoroalkyl substances, PFOA (perfluorooctanic acid), PFHxS (perfluorohexane sulfonic acid), PFNA (perfluorononanoic acid).

Industry Self-Regulation. Governmental regulations have slowed in areas where industry was self-regulated. As discussed above, 3M and DuPont voluntarily ended production of PFOS and PFOA in the early 2000s [42,43]. As shown in Figure 3, PFOS blood levels declined by 80%, and PFOA blood levels declined by 60% since these chemicals were phased out of production [44]. Although EPA is moving toward regulating these PFAS under the SDWA [201], because they are no longer being produced, states may be reluctant to invest resources in monitoring or remediating them. For example, the Oregon Department of Environmental Quality states that that “no major source of PFAS has been found in Oregon that would create regular exposure for Oregonians.” [202]

## 4. Discussion

There is a growing body of literature and data collection tools which document PFAS laws, policies, and regulations as they are proposed and passed; however there are no sources of data studying PFAS policy from a systems level, developing a conceptual framework for how PFAS policy is discussed, developed, and implemented. Our review of international-, U.S. national- and U.S. state-level policy and regulatory guidance shows a complex regulatory system both facilitated and challenged by a multitude of political, social, economic, and scientific feedback loops. The synthesizing of the current policy environment is a critical component to filling gaps in the scientific literature and building evidence-based regulatory requirements.

The regulation of PFOS and other PFAS has been part of the international dialogue for more than a decade [37,65,66,76]. The increasing scientific data documenting the widespread contamination and negative effects on human health, coupled with a rising social awareness, has prompted countries to meet the challenges of regulation at varying levels [76,85,88]. While the EU and Canada have established strong regulations focused on reducing and controlling the production and use of PFAS [76,81,84], other countries such as the United States and Australia have provided education and standards, leaving regulatory action to states and territories [64,88,89,96,97]. Furthermore, other countries such as Brazil and China continue to allow the production and use of some PFAS that are limited by the Stockholm Convention [73,74].

Our investigation found a deep interconnectedness between political willingness, social awareness, and scientific literature as both drivers of and challenges to PFAS policy implementation. The scientific study of PFAS has allowed for the categorization of PFAS into subgroups of long- and short-chain PFAS, as well as bans on compounds such as PFOA and PFOS [1,20,22,24,25]. More than two decades of laboratory study and environmental sampling shows PFOS and PFOA can have detrimental consequences on the physiology of animals, can accumulate in plants, and may have harmful health effects in humans [45,46,47,48,49]. Studies such as these have caught the attention of the legal community, resulting in high-profile lawsuits [125,203,204], which in turn created compelling movies [148,149] all of which have increased the social awareness and political pressure to respond and regulate PFAS within the environment. These reinforcing feedback loops drive the system towards change.

Simultaneously, the scientific literature that informs and supports the development of national and state-level policy does not always have an adequate specificity to support regulations. While PFAS have been categorized, there is no authoritative list of all PFAS compounds in use, hindering the ability of the interested parties to empirically study the health effects of all types of PFAS [1,7,20,21]. Although animal studies have shown that some PFAS compounds have deleterious effects [45,46,47,48], there remains the doubt that some of these effects are seen in humans. This is further complicated by the sheer volume of individual PFAS that have been used. It is not feasible to develop full toxicity assessments for each of the 4000–5000 PFAS compounds that have entered the global market. Although some have advocated for regulations targeting PFAS as a class [9,25,54,151], the current regulatory structure at the U.S. national-level requires chemical-specific data. The political will to regulate a compound that has documented benefits to society is weakened by what is deemed as lacking scientific evidence of harm. These restrictive feedback loops drive the system away from change.

Although the U.S. government has been slower to implement national-level policy than other governments, it has provided financial and technical support to states in evaluating PFAS levels. For some states, the slower pace of national-level policy initiates a reinforcing feedback loop in which what is perceived as a gap in national environmental regulation leads to the social mobilization and investment of local resources in support of a state-based action. For other states, the pace of national-level policy initiates a restrictive feedback loop, where clear lines of authority and insufficient technical expertise, combined with a lack of federal guidance to create challenges in developing and implementing PFAS policy.

One limitation of this research is that scarce information was available about attempts to implement PFAS regulations and policies in some U.S. states. Using a survey of states, ECOS identified 23 states which had not implemented any state-level PFAS guidelines [177]. Five of these states reported that the state had restrictions prohibiting them from setting guidelines for drinking water or groundwater guidelines that were more restrictive than those set by the federal government [177]. Although we have identified challenges to PFAS policy implementation for some states where policies were proposed, our knowledge about states in which policies were not proposed remains limited. Primary data collection such as stakeholder interviews may provide a more complete picture of the challenges these states face in implementing PFAS policies.

Another limitation of this study is that our review of the drivers of and challenges to PFAS policy implementation is limited to the U.S. Two of the three primary data sources used for this review focused on U.S.-based newspaper articles and business journals. Supplemental searches targeted U.S. state government websites, consumer group websites, and public comment dockets. With the absence of guidance of local stakeholders, we cannot be confident in our ability to identify comparable supplemental sources of information for non-U.S. countries.

Our focus on English language documents further limits the extent to which our findings about the drivers of and challenges to PFAS policy may be generalizable outside the U.S. Policymakers in other countries may face challenges in regulating the PFAS not discussed here, such as lower prioritization of environmental issues, lack of government accountability, and a lack of funding to support PFAS legislation. We encourage future research to explore the drivers of and challenges to PFAS policy in non-U.S. countries, including non-English speaking countries and developing countries. Such exploration should be guided by individuals who are knowledgeable about policymaking in the country (or countries) of focus, including the law-making process, appropriate sources of information, culturally meaningful search terms, and a deep understanding of the broader challenges that regulators face in that country.

## 5. Conclusions

National, state, and local policymakers and stakeholders face many challenges when developing policies for any complex system. Environmental and health-focused policies are impacted by many variables, including political will, social awareness, economic and scientific resource availability, and practical challenges. PFAS regulation faces the challenges of unclear lines of regulatory authority, challenges funding monitoring and remediation efforts, incomplete scientific evidence, and practical challenges. The balance between the perception of the benefits of PFAS on societal functions and the deleterious effects of PFAS on the environment and human health is influencing both national and state regulation. Policymakers and advocates should remain aware of the political, social, and scientific ecosystem influencing this rapidly changing landscape.

## Figures and Tables

**Table 1:** Inclusion and exclusion criteria. Documents were considered relevant if they met inclusion criteria 1 and were from a source described in inclusion criteria 2–5. Documents were considered not relevant if they met any of the exclusion criteria.

Inclusion Criteria	Exclusion Criteria
1. Documents contain information on PFAS laws, policies, and/or regulations.	1. PFAS-related documents that do not discuss PFAS laws, policies, or regulations.
2. Peer-reviewed scientific literature.	2. Information from unclear sources such as blog posts or social media content.
3. Government documents.	3. Documents not written in or translated to English.
4. Published gray literature such as news articles, trade journal articles, and interviews.	
5. Government or research organization website content.	

PFAS: per- and polyfluoroalkyl substances.

**Table 2:** Databases searched and search parameters used to identify relevant articles.

Database	Filter(s)	Search String
PubMed	English	((PFAS[Title] OR PFOA[Title] OR PFOS[Title] OR perfluoro[Title]) AND ((policy[Title] OR policies[Title] OR law[Title] OR laws[Title] OR legislation[Title] OR regulatory[Title] OR regulation[Title] OR bill[Title] OR bills)[Title])
SciTech Premium Collection	Title Field	(PFAS OR PFOA OR PFOS OR perfluoro) AND (policy or policies or law or laws or legislation or regulatory or regulation or bill or bills)
Business Source Complete	Title Field	(PFAS OR PFOA OR PFOS OR perfluoro) AND (policy or policies or law or laws or legislation or regulatory or regulation or bill or bills)

**Table 3:** Policy themes captured in literature search.

Variable	Description
Political	The likelihood of PFAS policies being implemented is a function of interconnected political factors, including the perceived need for regulation, collaboration among political leaders, and feedback loops where policy or a lack of policy at one level influences the policy implementation at another level.
Social	Awareness of PFAS-issues within a community, including awareness that is increased by the media, regulatory processes such as public forums and hearings, and lawsuits against PFAS manufacturers, drive the adoption of PFAS policies.
Economic	PFAS monitoring and remediation efforts are expensive. At the state-level, financial support from the federal government drives PFAS policy. In the absence of this support, states face significant challenges funding PFAS monitoring and remediation efforts.
Scientific	Toxicity data are required to support PFAS regulation in some cases. Even when such requirements are absent, it is easier to gain support for PFAS policies when there is compelling scientific evidence that these compounds have harmful effects. Thus, regulations are more likely to be implemented for legacy compounds than chemically similar compounds that are still in use.
Practical	PFAS policies and regulations require technical expertise to implement and many states do not have access to experts. Industry self-regulation may reduce the motivation of some states to enact PFAS policies and regulations.

PFAS—per- and polyfluoroalkyl substances.

**Table 4:** Variables and sub-variables that drive adoption of PFAS-related laws, policies, and regulations.

Variable	Description
Political	Perceived Regulatory Need. A lack of U.S. national regulatory guidance drives states to fill the gap with their own regulations (e.g., MCLs). Political Collaboration. Bipartisan support for PFAS policies. Political Leader Advocacy. The political party or leader in power prioritizes particular environmental policies/regulations without bipartisan consensus. Reinforcing Feedback Loops. PFAS regulations in one state build momentum to support regulations in other states.
Social	Social Awareness. Communities have been, or suspect that they have been, negatively impacted by PFAS exposure or voice concerns about exposure, either on the basis of their own health concerns or concerns about health equity.
Economic	Economic Support. Adequate financial resources to address PFAS regulations or remediation efforts are made available to policy makers.
Scientific	Health Impacts. A body of evidence to support regulation is available, clear, and compelling.

PFAS: per- and polyfluoroalkyl substances; MCL: Maximum Contaminant Levels.

**Table 5:** Variables and sub-variables that drive away from the adoption of PFAS-related laws, policies, and regulations.

Variable	Description
Political	Restrictive Legislative Feedback Loops. Lack of regulatory action at the U.S. national-level leads to inaction at the state-level. Regulatory Limitations. Some states are hesitant to set regulations in the absence of federal guidance and/or national regulations. Differing Priorities. Some states prioritize the regulation of other substances above PFAS regulation.
Economic	Economic Challenges. PFAS monitoring and remediation efforts are cost-prohibitive for some U.S. states.
Scientific	Scientific Ambiguity. Scientific evidence demonstrating harm from exposure to many PFAS is ambiguous, lacks specificity, or is unavailable.
Practical	Practical Challenges. Testing and monitoring for PFAS contamination and exposure presents technical challenges. Industry Self-Regulation. Voluntary removal of some PFAS from the market has reduced regulatory momentum.

## Supplementary Materials

The following are available online at https://www.mdpi.com/article/10.3390/ijerph182010900/s1, Database S1: PFAS Review Library. An Excel database containing the 550 data sources used to complete this review.
